# Impact of mental and physiological fatigue on attentional focus in basketball players: a systematic review

**DOI:** 10.3389/fpsyg.2025.1760814

**Published:** 2026-01-21

**Authors:** Chen Zhu, Xinao Li, Shujia Liu

**Affiliations:** 1 College of Physical Education and Health, Chongqing Three Gorges University, Chongqing, China; 2 School of Physical Education, Yuxi Normal University, Yuxi, China; 3 College of physical education and health, Geely University of China, Chengdu, China

**Keywords:** decision-making, perceptual–cognitive performance, performance, quiet eye, team sports

## Abstract

**Objectives:**

Mental and physical fatigue may impair basketball players’ attentional focus and performance, but experimental evidence is limited. Thus, the objective was to systematically review studies on the acute effects of mental and/or physical fatigue on attentional focus, visual search/quiet eye behavior, and performance in basketball tasks.

**Methods:**

Following PRISMA 2020, we searched PubMed, Scopus and Web of Science. Eligible studies experimentally manipulated fatigue in basketball players and assessed attentional focus (i.e., internal–external focus instructions/self-reported focus, and visual–attentional control indexed by gaze/quiet eye and visual search metrics) and basketball-specific performance (decision-making accuracy/response time and shooting outcomes). Two authors screened records, extracted data, and assessed risk of bias. Because of the small number of included studies, heterogeneity and incomplete reporting, findings were synthesized narratively and individual study effect sizes were interpreted descriptively rather than pooled.

**Results:**

Out of 492 records, five studies (*n* = 89 male players) were included, two on mental fatigue and three on physical fatigue, and no eligible study included female athletes, limiting generalizability to women’s basketball. Mental-fatigue protocols using a basketball videogame consistently impaired video-based decision-making and altered visual search; one study showed that anodal transcranial direct current stimulation attenuated these effects. Moderate physical fatigue showed inconsistent or trivial effects on shooting outcomes, whereas severe physical fatigue reduced free-throw accuracy and quiet eye duration, while external attentional focus partially protected shooting performance. All studies showed at least “some concerns” or higher risk of bias.

**Conclusion:**

Preliminary evidence suggests that both mental and physical fatigue disrupt attentional focus and can impair basketball performance. Larger, preregistered, and more ecologically valid trials are required. Registration: OSF (osf.io/6hz9p).

**Systematic review registration:**

https://doi.org/10.17605/OSF.IO/Z4F2N, identifier osfio/z42n.

## Introduction

In fatigue research, multiple explanatory frameworks have been proposed. Beyond peripheral/neuromuscular accounts, the Central Governor Model (Noakes, 2007) conceptualizes fatigue as an integrative, brain-mediated regulatory process that constrains performance to protect homeostasis, thereby shaping pacing and effort allocation (Noakes, 2012). Resource depletion accounts propose that prolonged cognitive demands may reduce available executive resources and impair subsequent attentional control and decision-making (Wu et al., 2024). Thus, fatigue is a multifaceted phenomenon in sport, encompassing both physiological and mental components that interact to constrain athletes’ ability to sustain high-quality performance over time (Van Cutsem et al., 2017). Mental fatigue is classically defined as a psychobiological state elicited by prolonged, demanding cognitive activity, producing subjective feelings of tiredness, reduced motivation, and impairments in executive control and decision-making (Marcora et al., 2009). In parallel, physiological fatigue reflects exercise-induced reductions in force-generating capacity arising from central and peripheral mechanisms along the neuromuscular pathway, with important consequences for technical execution and perceptual-cognitive functioning during sport-specific tasks (Li et al., 2025). A growing body of evidence indicates that mental fatigue impairs sport-specific psychomotor performance, slowing response speed, degrading accuracy in complex skills, and exacerbating perceived exertion across endurance and team sports (Habay et al., 2021). Recent narrative and systematic reviews further suggest that mental fatigue is particularly detrimental for tasks requiring sustained selective attention, rapid decision-making, and precise visuomotor control, highlighting attentional processes as a plausible mechanism underlying performance decrements in athletes (Wu et al., 2024).

Basketball is a high-intensity, intermittent team sport that places simultaneous demands on repeated sprinting/jumping, rapid perceptual–cognitive processing, and continuous tactical scanning (Stojanovic et al., 2018; Martinho et al., 2025). These combined constraints make basketball a particularly relevant context for examining how fatigue disrupts attentional control and gaze behavior (Luo et al., 2025), because players must repeatedly perceive evolving configurations (teammates/opponents/space) while executing precision skills such as passing and shooting under time pressure. Accordingly, fatigue-related decrements in attentional focus and visual search may translate quickly into degraded decision quality and skill execution in basketball-specific tasks (Dong et al., 2022).

Attentional focus, defined as the direction of an athlete’s conscious attention toward internal bodily sensations or external movement effects and environmental cues, is a central construct linking cognition, perception, and motor performance in sport (Chua et al., 2021). Experimental and meta-analytic work shows that an external focus (e.g., attention to the ball trajectory or the basket) generally enhances movement effectiveness and efficiency compared with an internal focus (e.g., attention to limb movements), supporting the constrained-action hypothesis that externally directed attention promotes more automatic motor control (Chua et al., 2021). Importantly, attentional focus is not only a determinant of skill execution but also modulates perceived exertion and fatigue, with externally focused instructions reducing ratings of perceived effort and, in some contexts, attenuating performance fatigability relative to internal focus (Lohse and Sherwood, 2011). Visual attention in aiming tasks is often indexed through gaze behavior, particularly the “quiet eye” (QE) period (the final fixation or tracking gaze on a task-relevant location immediately before movement initiation) where longer QE durations and more economical fixation patterns are consistently associated with superior performance in interceptive and aiming sports (Lebeau et al., 2016). These converging findings suggest that fatigue-related disruptions in attentional focus and gaze regulation may represent a key pathway through which both mental and physiological fatigue impair sport performance (Sun et al., 2021).

Basketball is a high-intensity intermittent team sport that places substantial physiological, technical, tactical, and perceptual-cognitive demands on players throughout match play (McInnes et al., 1995). Time–motion and heart-rate analyses show that players frequently operate at intensities above 85% of maximal heart rate, interspersing repeated accelerations, decelerations, jumps, and changes of direction with brief recovery periods, thereby generating considerable cumulative neuromuscular fatigue during competition (Petway et al., 2020). Within this context, effective performance in passing, shooting, and defensive and offensive decision-making is critically dependent on the ability to selectively attend to dynamic spatial–temporal cues from teammates, opponents, and the ball under severe time pressure and situational constraints (Piras, 2024). Gaze-tracking studies in basketball indicate that successful free throws and jump shots are characterized by longer quiet eye durations, fewer but more stable fixations on the rim or backboard, and more consistent head–eye coordination compared with missed shots or less-skilled performers (Vickers et al., 2019). A recent systematic review of visual attention in basketball shooting confirms that longer QE durations and more economical fixation patterns are reliably associated with higher shooting accuracy across free throws and jump shots, reinforcing visual–attentional control as a critical determinant of shooting performance in this sport (Sirnik et al., 2022).

Despite this strong linkage between visual–attentional control and shooting success, basketball players must sustain optimal attentional focus under considerable physical and psychological strain, including the progressive build-up of physiological fatigue and the mental fatigue induced by sustained tactical engagement, emotional regulation, and decision-making over the course of training and competition (Wu et al., 2024). Experimental work simulating match-related physiological fatigue via repeated-sprint or intermittent-running protocols has shown decrements in basketball-specific passing and shooting accuracy following fatigue, even in skilled players, suggesting that neuromuscular fatigue compromises fine motor control and coordination in key technical skills (Lyons et al., 2006). Studies focusing on shooting have reported that exertion manipulations reduce free-throw and field-goal accuracy and alter kinematic parameters such as release height, entry angle, and upper-limb joint velocities, supporting the idea that fatigue disrupts the biomechanics underlying accurate shooting (Slawinski et al., 2018). Building on this work, recent kinematic and performance analyses under game-like fatigue indicate that progressive physiological load and combined fatigue–defensive pressure scenarios substantially modify three-point jump-shot mechanics and can impair shooting effectiveness, especially at longer distances from the basket (Bourdas et al., 2024). Importantly, emerging evidence suggests that these fatigue-related performance decrements are not purely mechanical: exertion has been shown to shorten quiet eye durations, increase gaze dispersion, and degrade visual control strategies during shooting, indicating that physiological fatigue may directly perturb visual attention and attentional focus in basketball tasks (Zwierko et al., 2018).

In parallel, a rapidly growing literature has examined the effects of mental fatigue on sport-specific skills, highlighting its particular relevance for decision-rich, open-skill team sports such as basketball (Sun et al., 2021). A systematic review consistently report that mental fatigue impairs sport-specific psychomotor performance, including technical execution and tactical decision-making, with larger effects observed for offensive skills that demand rapid integration of perceptual information and response selection (Habay et al., 2021). Within basketball, a dedicated systematic review concluded that mental fatigue negatively affects technical indicators (e.g., shooting percentages, turnovers), cognitive performance, and decision-making, while evidence on physical and tactical outcomes remains relatively scarce (Cao et al., 2022). Experimental studies in basketball players have demonstrated that mental fatigue induced by prolonged cognitively demanding tasks or sport-based video games impairs free-throw accuracy, slows and degrades passing and tactical decision-making, and alters visual search behavior—typically increasing the number of fixations and reducing their duration, consistent with less efficient attentional allocation (Fortes et al., 2025). Recent work combining manipulations of physiological fatigue and attentional focus instructions shows that external focus can partly counteract fatigue-related declines in shooting accuracy, pointing to attentional focus as a potential moderating mechanism at the intersection of fatigue and performance in basketball (Luo et al., 2025).

Some reviews have synthesized related but distinct segments of this literature, yet none has specifically integrated mental and physiological fatigue effects on attentional focus in basketball. Broad sport-science reviews on mental fatigue show robust negative effects on sport-specific motor and psychomotor performance but aggregate across sports, skills, and outcomes without isolating attentional focus and gaze-related measures in basketball contexts (Sun et al., 2021). Attentional focus is operationalized through conscious focus direction (internal vs. external focus instructions/self-reported focus) and visual–attentional control indexed by gaze behavior and visual search (as fixation number/duration and quiet eye timing/duration) (Lohse and Sherwood, 2011; Lebeau et al., 2016; Vickers et al., 2019; Chua et al., 2021). These indicators are treated as functionally linked to performance because internal–external focus modulates movement control, while gaze stability and efficient cue sampling are proximal determinants of shooting accuracy and decision quality in basketball tasks. Systematic overviews of basketball fatigue have primarily emphasized changes in shooting accuracy and kinematics under physical and, more recently, mental fatigue, with little attention to how these forms of fatigue influence attentional focus constructs such as internal–external focus, quiet eye, or visual search strategies (Li et al., 2025). Conversely, systematic reviews of visual attention and quiet eye in basketball shooting have confirmed strong associations between gaze behavior and shooting success but have treated fatigue only as one of several contextual modifiers, without differentiating mental from physiological fatigue or systematically characterizing fatigue protocols and their cognitive consequences (Sirnik et al., 2022). To our knowledge, no prior systematic review has comprehensively synthesized evidence on how both mental and physiological fatigue affect attentional focus (indexed by focus instructions, gaze behavior, e.g., quiet eye, and visual search patterns) and related decision-making processes in basketball players across competitive levels, task conditions, and fatigue paradigms, nor examined methodological quality and risk of bias across this emerging body of work (Habay et al., 2021). One reason this gap persists is that the literature is methodologically siloed since mental-fatigue work in team sports often prioritizes decision accuracy/response time, whereas physiological-fatigue work in basketball has tended to prioritize shooting accuracy/kinematics, with gaze and attentional focus treated as secondary or omitted outcomes (Smith et al., 2016; Trecroci et al., 2020). Parallel evidence streams in other open-skill sports shows that mental fatigue can degrade sport-specific decision-making and technical/tactical behavior, providing a useful comparative baseline for interpreting basketball findings (Nuño et al., 2016). In addition, we highlight methodological controversies likely to influence inference in this area, including the generalizability of laboratory fatigue paradigms (e.g., stationary cycling) to basketball-specific movement demands and the strength and reproducibility of neuromodulation (tDCS) effects on perceptual–cognitive performance (Chinzara et al., 2022; Fortes et al., 2022).

Integrating mental and physiological fatigue is particularly warranted in basketball because match play co-produces sustained cognitive load (tactical scanning, decision-making, emotional regulation) alongside repeated high-intensity efforts that generate neuromuscular and cardiorespiratory strain (Marcora et al., 2009; Angius et al., 2022). These fatigue modalities may converge on shared attentional outcomes (e.g., gaze stability, cue sampling) while operating through partly distinct mechanisms, making a unified synthesis essential for identifying robust versus fatigue-type-specific effects and for informing practical countermeasures. Accordingly, the objectives of the present systematic review are: (i) to synthesize and critically appraise experimental and quasi-experimental evidence on the impact of mental and physiological fatigue on attentional focus and related visual-cognitive outcomes in basketball players of any age, competitive level or sex; (ii) to examine how these effects vary according to player characteristics, task constraints, and fatigue induction protocols; and (iii) to identify methodological limitations and knowledge gaps to guide future research and inform evidence-based training and competition strategies aimed at preserving attentional focus and performance under fatigue in basketball.

## Methods

This systematic review was conducted and reported in accordance with the PRISMA 2020 statement and its expanded checklist for systematic reviews (Page et al., 2021). The protocol, including the review question, eligibility criteria, and planned methods for data collection and synthesis, was defined a priori and prospectively registered in the OSF (osf.io/6hz9p; 02/12/2025).

### Eligibility criteria

Eligibility criteria were structured according to a Population–Exposure/Intervention–Comparator–Outcome–Study design (PECOS) framework adjusted to the review question on fatigue and attentional focus in basketball. We included studies involving basketball players of any sex, age group (youth to adult), and competitive level (recreational to professional), provided that participants were engaged in basketball-specific tasks (e.g., shooting, passing, decision-making, small-sided games). Experimental or quasi-experimental studies that induced mental fatigue (e.g., through prolonged cognitively demanding tasks) and/or physiological fatigue (e.g., via intermittent running, repeated-sprint protocols, game-based drills, or match play) and assessed their acute effects on attentional focus or related visual–cognitive outcomes during basketball tasks were eligible. Comparators included non-fatigued or baseline conditions, lower-intensity/control tasks, sham or low-demand cognitive tasks, or alternative fatigue conditions differing in type, intensity, or duration.

Primary outcomes were measures of attentional focus and visual–attentional control, operationalized as: (i) attentional focus instructions or self-reported focus (e.g., internal vs. external focus); (ii) gaze behavior and quiet eye parameters (e.g., QE onset/offset, QE duration, number and duration of fixations, fixation location); and (iii) visual search behavior (e.g., scan patterns, saccade metrics, visual pickup of task-relevant cues). Secondary outcomes included basketball-specific performance indicators linked to attentional control (e.g., free-throw or field-goal accuracy, passing accuracy, decision-making accuracy or response time, turnovers), as well as psychophysiological or perceptual measures that might mediate fatigue effects on attention (e.g., rating of perceived exertion, subjective mental fatigue, mental effort). We included randomized controlled trials, randomized or non-randomized crossover or repeated-measures experiments, and other controlled intervention or exposure studies (e.g., controlled pre–post designs) that allowed within- or between-group comparisons of fatigued versus non-fatigued conditions. Purely observational studies without a defined fatigue manipulation were excluded. Reports were eligible regardless of publication status and year. No language restrictions were applied, and non-English articles were translated using bilingual collaborators.

### Information sources and search strategy

A comprehensive literature search was conducted in the following electronic databases from inception to 03/12/2025: PubMed, Web of Science Core Collection, and Scopus. For each database, we adapted a core search strategy combining controlled vocabulary and free-text terms related to basketball, fatigue, and attentional/visual processes. The searches were made for the title/abstracts and key-words or topic in the case of Web of Science database. The following search strategy was implemented:

[Basketball OR basketballer OR basketballers] AND [“mental fatigue” OR “physical fatigue” OR “physiological fatigue” OR fatigue OR fatiguing OR fatigued OR effort OR exhausted OR exhaustion] AND [attention OR “attentional focus” OR “quiet eye” OR gaze OR “visual search” OR “decision making” OR “visual attention” OR “visual information” OR “visual fixation” OR shooting].

To identify additional studies, we consulted Google Scholar for forward citation tracking of key included articles. Reference lists of all included studies and relevant prior reviews on mental fatigue, physiological fatigue, and visual attention/quiet eye in basketball and other invasion games were hand-searched to identify further eligible records (Page et al., 2021).

### Selection process

All records retrieved from the searches were imported into a reference management program (Endnote). The study selection process followed PRISMA 2020 guidance (Page et al., 2021). Two reviewers independently screened titles and abstracts against the eligibility criteria, classifying records as “include,” “exclude,” or “uncertain.” Full-text articles were then retrieved for all records classified as “include” or “uncertain,” and the same two reviewers independently assessed full texts for final inclusion. Any disagreements at either stage were resolved through discussion. When consensus could not be reached, a third reviewer arbitrated. Reasons for exclusion at full-text stage (e.g., non-basketball population, no fatigue manipulation, no attentional or visual outcomes, inadequate design) were recorded and reported in a PRISMA flow diagram alongside the number of records identified, screened, excluded, and included.

### Data collection process

Data extraction was conducted using a standardized, pilot-tested extraction form developed specifically for this review. Two reviewers independently extracted data from each included study, working in duplicate and blinded to each other’s entries. After independent extraction, forms were compared and any discrepancies were resolved through discussion, with arbitration by a third reviewer when needed. When information was missing, unclear, or inconsistent across multiple reports of the same study, study authors were contacted by email (up to two attempts) to obtain or verify the data. Where necessary, numerical data presented only in figures were extracted using digital measurement software (WebPlotDigitizer v4.8), and, when articles were not in English, extraction was performed using translated versions that were independently checked by at least one bilingual reviewer. Pre-specified decision rules were applied to harmonize data across multiple reports from the same underlying study (e.g., prioritizing peer-reviewed full articles over conference abstracts when overlapping data were present).

### Data items

For each study, we extracted detailed information on: (i) study characteristics (authors, year, country, setting, study design, publication type); (ii) participant characteristics (sample size, sex distribution, age, competitive level, inclusion/exclusion criteria); (iii) details of the fatigue manipulation, specifying whether it targeted mental fatigue, physiological fatigue, or both, including task type (e.g., Stroop, n-back, video-based tactical tasks, intermittent running or repeated-sprint protocols, small-sided games), intensity, duration, work–rest ratios, and timing relative to the basketball task; and (iv) details of the basketball tasks and contextual constraints (e.g., free throws vs. jump shots, passing or decision-making drills, 1v1 or small-sided games, defensive pressure, distance to basket). We also extracted information on attentional focus manipulations or assessments (e.g., standardized internal vs. external focus instructions, self-report scales of focus, manipulation checks), the presence of coaching or verbal feedback, and whether attentional focus was manipulated orthogonally to fatigue (e.g., factorial designs).

Regarding outcomes, we collected all available data compatible with the predefined outcome domains and time frames. For visual–attentional outcomes, we extracted gaze metrics (quiet eye onset/offset, QE duration, number and duration of fixations, fixation locations, saccade frequency and amplitude, measures of gaze dispersion) and visual search indices as reported. For attentional and decision-related outcomes, we extracted behavioral performance (e.g., decision accuracy, response times, choice selection) and, where available, process-tracing metrics (e.g., cue utilization indices). For technical performance outcomes, we extracted measures such as shooting percentage, free-throw accuracy, field-goal percentage, passing accuracy, turnovers, or composite technical performance scores. When multiple time points or conditions were reported, we prioritized the assessment closest to the end of the fatigue manipulation and the corresponding non-fatigued comparator. Where several relevant outcomes or time points existed within a domain, we predefined decision rules (e.g., prioritizing primary outcomes as identified by the authors or the most basketball-specific task) and documented any deviations.

### Risk of bias assessment

Risk of bias was assessed for each included study at the study and (where appropriate) outcome level, using design-specific tools. For studies described as randomized controlled trials (either parallel-group RCTs or randomized crossover/within-subject designs with an explicit random allocation sequence and allocation mechanism) we used the revised Cochrane risk-of-bias tool for randomized trials (RoB 2) (Higgins et al., 2020). In these studies, we evaluated bias arising from the randomization process, deviations from intended interventions, missing outcome data, measurement of the outcome, and selection of the reported result, following the signaling questions and decision algorithms provided in the RoB 2 guidance. Each domain was judged as “low risk of bias,” “some concerns,” or “high risk of bias,” and an overall risk-of-bias judgment for each result was derived accordingly.

For non-randomized experimental and quasi-experimental designs, we used the Risk Of Bias In Non-randomized Studies of Interventions (ROBINS-I) tool (Sterne et al., 2016). This category included studies with controlled pre–post designs without random allocation, non-randomized crossover or repeated-measures experiments, studies in which allocation to fatigue condition was determined by practical or researcher constraints (e.g., fixed order of conditions, allocation by team/coach decision), and any design where randomization was either absent or insufficiently described to be judged as robust. For single-group pre–post exertion designs without a concurrent control, ROBINS-I was applied against a hypothetical target trial comparator, and we therefore interpreted domain ratings conservatively, emphasizing bias due to confounding, time-varying factors, and selective reporting as primary threats. ROBINS-I assesses bias in relation to a hypothetical target randomized trial, across seven domains: bias due to confounding, selection of participants into the study, classification of interventions, deviations from intended interventions, missing data, measurement of outcomes, and selection of the reported result. Each domain was judged as “low,” “moderate,” “serious,” or “critical” risk of bias (or “no information”), and these domain-level ratings were combined to provide an overall risk-of-bias judgment for each study and outcome.

Two reviewers independently applied the appropriate tool (RoB 2 or ROBINS-I) to each study, after calibration on a subset of studies to ensure consistent interpretation of the signaling questions and domain-level judgments. Disagreements were resolved through discussion, and when consensus could not be achieved, a third reviewer acted as arbiter.

### Effect measures and synthesis methods

For continuous outcomes (e.g., QE duration, number of fixations, shooting accuracy expressed as a percentage, decision-making accuracy), the primary effect measure was the standardized mean difference (SMD, Hedges g) between fatigued and non-fatigued (or lower-fatigue) conditions, with 95% confidence intervals. When not directly reported, means and standard deviations were derived from other summary statistics (e.g., standard errors, confidence intervals, F-values) using established formulae. For crossover or repeated-measures designs, we preferentially used paired analyses and, when within-subject correlations were not reported, we applied reasonable imputed correlation coefficients based on similar studies and explored their impact in sensitivity analyses where meta-analysis was feasible. For dichotomous outcomes (e.g., success vs. failure of a shot when reported only as counts), risk ratios or odds ratios with 95% confidence intervals were calculated.

Given the anticipated methodological and clinical heterogeneity across studies in fatigue protocols, attentional focus manipulations, and outcome measures, we planned a primarily narrative synthesis structured around key conceptual groupings. Studies were grouped for synthesis according to (i) type of fatigue (mental vs. physiological vs. combined), (ii) type of attentional/visual outcome (attentional focus instructions/self-report vs. gaze/QE vs. visual search vs. decision-making vs. technical performance), (iii) task type (e.g., free-throw vs. jump-shot vs. passing vs. decision-making drills vs. game-like tasks), and (iv) competitive level (e.g., youth, semi-elite, elite).

## Results

### Study selection

**FIGURE 1:**
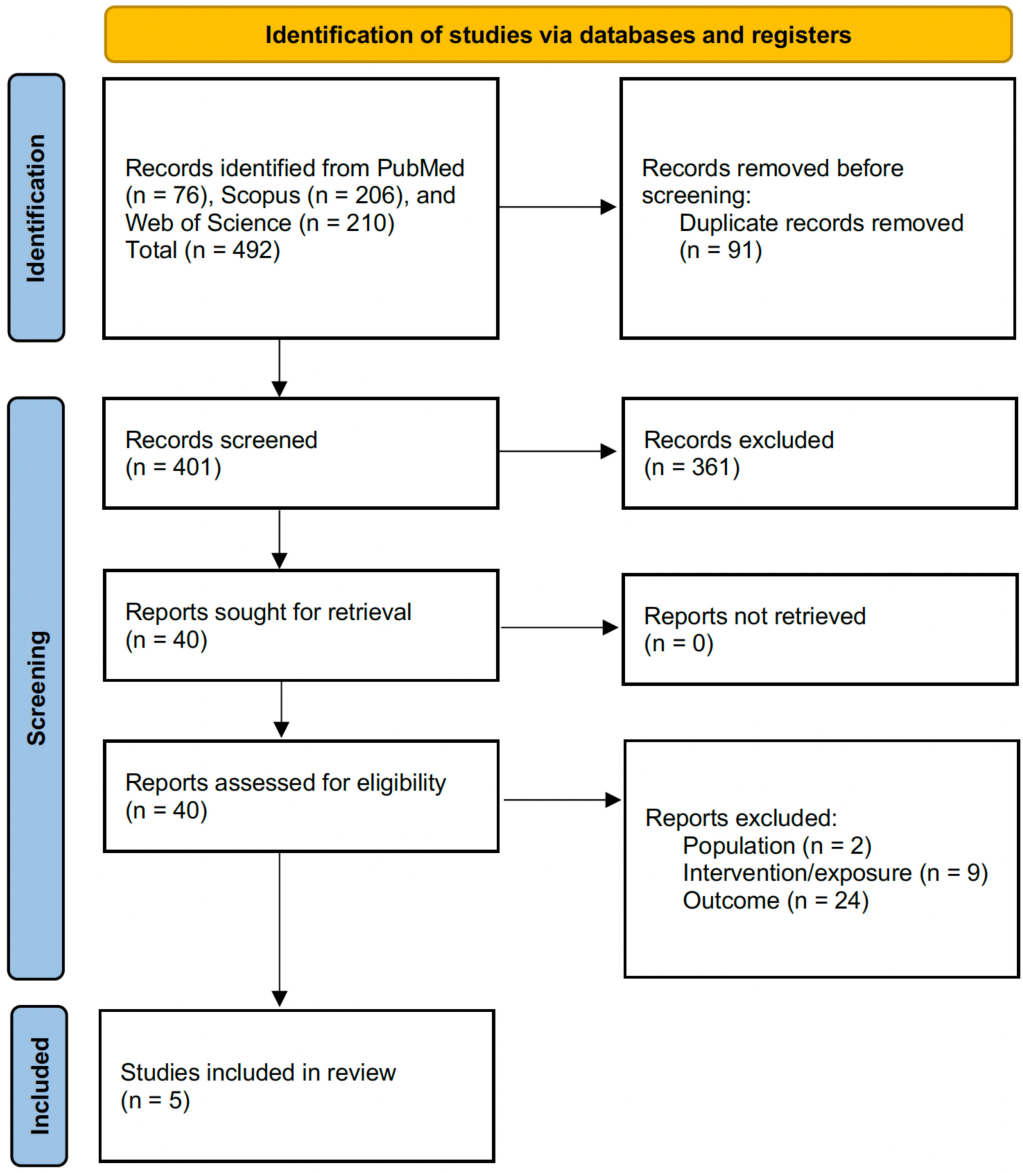
PRISMA flow diagram.

A total of 492 records were identified through database searching (PubMed, *n* = 76; Scopus, *n* = 206; Web of Science, *n* = 210). After removal of 91 duplicate records, 401 records remained for title and abstract screening. Of these, 361 records were excluded as not meeting the inclusion criteria. The full texts of 40 reports were assessed for eligibility, and 35 were excluded for the following reasons: ineligible population (*n* = 2), ineligible intervention/exposure (*n* = 9), and ineligible outcomes (*n* = 24). No reports were unobtainable. In total, 5 studies met all inclusion criteria and were included in the review (see Figure 1).

### Study characteristics

Five experimental studies met the inclusion criteria and are summarized in Table 1. All samples comprised male basketball players (*n* = 10–30) ranging from sub-elite/university to professional and national-level competitors, with mean ages between 20 and 25 years. None of the eligible experimental studies included female athletes, representing a critical evidence gap and limiting generalizability of the current conclusions. Most studies used randomized, within-subject crossover designs (including counterbalanced and double-blind comparisons), with one single-group repeated-measures study (Zwierko et al., 2018). Across studies, appropriate control conditions were included (e.g., sham stimulation, low-demand video viewing, no-fatigue warm-up only, or seated rest). Primary outcomes focused on attentional and perceptual–cognitive performance, including video-based 5-on-5 decision-making tasks with eye-tracking, free-throw shooting under internal vs. external focus instructions, and oculomotor measures such as quiet-eye duration and gaze behavior (number and duration of fixations on the hoop and other task-relevant areas).

**TABLE 1:** Study characteristics.

Study	N and sex	Age (mean ± SD)	Competitive level	Study design	Fatigue condition employed	Fatigue protocol (how fatigue was implemented)	Control condition	Measure of attentional focus/primary outcome (specific test)
Fortes et al. (2022)	20 male	24.8 ± 4.2 years	Professional male players, Brazilian National Basketball League (2nd division), ≥ 3 years at this level	Randomized, double-blind, counterbalanced crossover {anodal transcranial direct current stimulation (a-tDCS) vs. sham], repeated baseline/post measures	Mental fatigue	60-min continuous play of NBA Live 19 (PS4) in pairs on a 48′′ TV to induce sustained cognitive load; in the active condition, 2 mA anodal tDCS over MT/V5 (CP5–Oz montage) applied for 30 min during gaming	Sham tDCS (same montage, current off after ∼30 s) during the same 60-min videogame (non-stimulation control under similar mental load)	Perceptual–cognitive/attentional: Video-based 5-on-5 basketball decision-making task (first-person clips; % correct decisions and response time) with head-mounted eye-tracking to quantify gaze behavior (number/duration of fixations on ball, teammates, opponents, hoop, space); plus a visuomotor Fitlight task while dribbling (reaction time and accuracy)
Fortes et al. (2025)	16 male	24.3 ± 4.1 years	Professional male players, 2nd division Brazilian National Basketball League; national-level, ≥ 3 years at this level	Randomized, counterbalanced crossover with two conditions (mental-fatigue videogame vs. control video) and pre/post measures	Mental fatigue	60-min continuous play of NBA Live 19 (PS4) in pairs; high cognitive demand mental-fatigue protocol, validated via EEG frontal theta, Stroop performance and VAS mental-fatigue ratings	60-min viewing of emotionally neutral Olympic/coaching documentaries on a 48′′ screen in pairs (low cognitive demand; no game play)	Primary: Basketball-specific video-based 5v5 decision-making task (first-person clips; verbal choice of action, scored for decision accuracy and response time). Attentional focus measure: Eye-tracking during the decision task (number and duration of fixations on task-relevant areas)
Luo et al. (2025)	30 male	20.1 ± 0.3 years	Collegiate basketball players at Beijing Sport University; all First-Class Athletes (national classification)	Within-subject, randomized, counterbalanced crossover (3 fatigue levels × 2 attentional-focus conditions); all players completed all conditions in separate sessions	Physiological/physical fatigue	Three fatigue conditions: No fatigue: standardized warm-up only, then seated rest (∼10 min). Moderate fatigue: ∼5-min protocol with repeated 20 m shuttle runs, continuous jumps, and rapid shooting drills, targeting RPE ∼13, HR ∼150 bpm, lactate ∼5 mmol⋅L^–1^. Severe fatigue: ∼10-min high-intensity protocol with more shuttles, jumps and layups, targeting RPE ∼17, HR ∼175 bpm, lactate ∼9 mmol⋅L^–1^. HR, RPE, HRV and lactate used to verify and maintain fatigue level.	No-fatigue session (warm-up + seated rest, no additional exercise) served as the physiological control; same free-throw protocol and timing, with low HR, RPE and lactate confirming minimal fatigue	Attentional manipulation/primary performance: Free-throw shooting under external-focus vs. internal-focus instructions. In each fatigue condition, players attempted 20 regulation free throws under external focus (e.g., focus on hoop/ball flight) and 20 under internal focus (e.g., focus on hand/wrist motion). Primary outcome = free-throw performance under these attentional-focus conditions across fatigue levels
Wilson et al. (2018)	10 male	19–28 years; 22.5 ± 2.6 years	Experienced male players from university and local teams (amateur/sub-elite)	Randomized, within-subject crossover with four conditions (rest, moderate-, heavy-, severe-intensity cycling), each on a separate visit	Physiological/physical fatigue	After a baseline ramp test to determine VO2peak and gas-exchange threshold, players completed 10-min interventions on a cycle ergometer: REST (seated, no pedaling), MOD (∼80% GET), HEV (40%Δ between GET and VO2peak), SEV (65%Δ until exhaustion). Immediately afterwards (∼20–25 s) they performed post-intervention free throws. Graded HR responses confirmed increasing physiological stress	The rest condition (10-min seated rest on ergometer) served as the non-exercise control; pre-intervention free throws in each visit also acted as individual non-fatigued baselines	Attentional focus measure (Quiet Eye): QE duration during free-throw shooting, recorded with a mobile eye tracker (30 Hz). QE defined as the final fixation ≥ 100 ms within 1° of the target (rim/backboard/net) starting before elbow extension and ending when gaze deviated > 1° for > 100 ms. Primary outcome = mean QE duration pre vs. post in each exercise condition
Zwierko et al. (2018)	13 male	20.9 ± 2.4 years	Top two Polish National Basketball League divisions; ∼11 years high-level experience	Single-group, within-subject repeated-measures design (pre- vs. post-exertion shots in one session; no randomized between-condition groups)	Physiological/physical exertion	S2P60 (Two-Point Shooting During 60 s Fatigue test): After warm-up and baseline free throws, players performed 60 s of continuous two-point jump shooting while sprinting between six cones around the key, receiving passes and shooting as many jump shots as possible. HR was monitored; repeated-sprint ability decrement (SDEC ≈ 4%) confirmed substantial physical fatigue	Pre-exertion free throws (two attempts before S2P60) served as the low-fatigue control for comparison with post-exertion free throws; no separate non-exercise day was included	Primary attentional/oculomotor outcome: Gaze behavior recorded with SMI ETG 2 w mobile eye-tracker (60 Hz) during the motor phase of free throws and two-point jump shots. Fixation frequency and duration on the basket/hoop/backboard were quantified and compared pre- vs. post-fatigue, indexing visual attentional control under exertion

Control conditions differed across fatigue modalities. Mental-fatigue studies typically used time-matched low-demand video viewing controls, which control for duration and setting but may introduce differences in boredom/arousal and engagement. Physical-fatigue studies commonly used seated rest or low-load baselines, which better isolate exercise-induced physiological strain but do not control for cognitive engagement.

### Risk of bias in studies

Across the included experiments, most studies were randomized, controlled crossover lab designs in small samples of trained basketball players (Wilson et al., 2018; Fortes et al., 2022, 2025; Luo et al., 2025) and consequently showed overall “some concerns” rather than low risk of bias. In these four trials, the most recurrent issues were limited reporting of the randomization process and allocation concealment and the absence of trial registration or a pre-published analysis plan, despite multiple potentially reportable outcomes and analytic choices in cognitive performance, eye-tracking and physiological data. By contrast, the domains for deviations from intended interventions, missing data and outcome measurement were generally at low. Moreover, the primary performance outcomes were objective and instrument-based minimizing the scope for observer bias even when assessors were not explicitly blinded. In contrast, a pre–post exertion study (Zwierko et al., 2018), which lacked random allocation or a concurrent control condition and offered limited information on the completeness of gaze data, was judged at high overall risk of bias, driven by non-randomized design and additional concerns about possible unreported missing data and selective reporting within a rich set of eye-movement outcomes.

Importantly, the observed effects should be interpreted in light of these biases and small samples. The single-group pre–post exertion study judged at higher risk of bias reported pronounced gaze-pattern changes but limited performance change, whereas the crossover trials (mostly assessed as some concerns) generally reported more consistent condition effects on decision-making or shooting under severe fatigue. Overall, effect magnitude did not map cleanly onto risk-of-bias category, reinforcing the need for larger, preregistered trials with transparent outcome reporting (Table 2).

**TABLE 2:** RoB-2 assessment for the included studies.

Study	Bias from randomization process	Bias due to deviations from intended interventions	Bias due to missing outcome data	Bias in measurement of the outcome	Bias in selection of the reported result	Overall risk of bias
Fortes et al. (2022)	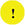	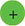	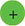	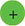	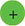	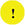
Fortes et al. (2025)	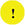	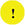	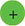	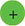	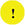	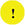
Luo et al. (2025)	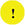	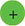	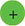	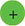	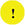	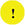
Wilson et al. (2018)	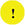	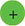	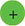	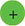	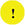	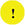
Zwierko et al. (2018)	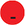	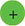	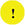	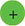	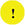	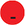

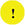
: low risk; 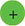: some concerns; 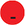: high risk.

### Results of the individual studies

Table 3 presents the visual search and gaze-behavior outcomes that accompany these performance changes, drawing on four of the five studies. It summarizes how mental fatigue, with or without transcranial direct current stimulation (tDCS), modifies the number and duration of fixations during complex decision-making in dynamic video clips (Fortes et al., 2022, 2025), and how physiological stress alters Quiet Eye duration and gaze patterns in free-throw shooting and jump shots (Wilson et al., 2018; Zwierko et al., 2018).

**TABLE 3:** Attentional focus, visual search/gaze behavior outcomes.

Study	Task and gaze outcome(s)	Main findings	Effect estimates and direction of effect
Fortes et al. (2022)	Basketball decision-making task performed under anodal transcranial direct current stimulation (a-tDCS) vs. sham after mental-fatigue videogame. Mobile eye-tracking recorded gaze to task-relevant areas (ball, teammates, opponents, hoop, open space). Outcomes: number of fixations and mean fixation duration (ms) across clips.	Following mental-fatigue induction, the sham condition showed a reduced number of fixations and increased fixation duration, indicating a slower, less flexible visual search pattern. Under a-tDCS, the number and duration of fixations were largely maintained, consistent with preserved search efficiency.	Significant Condition × Time interactions for both number of fixations and fixation duration [e.g., *F*(2, 38)≈22.7 for fixations; *F*(2, 38)≈50.2 for fixation duration; both *p* = 0.001, partial η^2^≈0.13–0.22], indicating that visual search deteriorated under sham but not under a-tDCS.
Fortes et al. (2025)	Decision-making task after VID (mental-fatigue videogame) vs. CON (control video), with baseline and post-tests. Gaze outcomes: number of fixations and fixation duration (ms) averaged across 20 clips.	From baseline to post, VID produced a decrease in number of fixations (∼9% reduction), whereas CON showed minimal change. Fixation duration did not change meaningfully in either condition.	Number of fixations: Condition × Time interaction *F*(1, 15) = 4.01, *p* = 0.03, partial η^2^ = 0.05—reduced number of fixations only after VID. Fixation duration: no significant main or interaction effects (all p > 0.05).
Wilson et al. (2018)	Free-throw task (10 shots pre- and post-intervention) performed after REST, MOD, HEV, or SEV cycling. Gaze outcome: Quiet Eye (QE) duration (ms)—final fixation on the target before and during the extension phase.	In the SEV condition, QE duration decreased from 1459 ± 865 ms to 808 ± 558 ms (∼45% reduction). Changes in QE in REST and MOD were minimal; HEV showed a smaller, non-significant decrease.	QE ANOVA: trend for main effect of time *F*(1, 9) = 5.10, *p* = 0.05; significant Condition × Time interaction *F*(3, 27) = 2.97, *p* = 0.049. Only SEV showed a significant pre–post decrease (*p* = 0.001). Regression analysis across conditions: change in QE explained ∼34% of variance in free-throw performance change [*F*(1, 28) = 19.53, *p* < 0.01, *R*^2^ = 0.339].
Zwierko et al. (2018)	Free throws (pre/post exertion) and two-point jump shots during S2P60 fatigue test. Gaze outcomes during the motor phase: (i) fixation frequency (number per shot), (ii) mean fixation duration (ms), (iii) total fixation duration.	Collapsed across exertion, free throws showed more and longer fixations than jump shots: fixation frequency 3.00 ± 1.42 vs. 2.29 ± 0.71; mean fixation duration 797.70 ± 508.77 vs. 355.55 ± 124.93 ms. After exertion, free throws showed increased fixation frequency (2.27 ± 1.11 → 3.73 ± 2.01) and reduced mean fixation duration (1002.09 ± 678.74 → 681.22 ± 361.39 ms); total fixation duration unchanged.	Free throw vs. jump shot: more fixations (*Z* = 2.06, *p* = 0.039) and longer fixations (*Z* = 3.18, *p* = 0.001) in free throws. Effect of exertion on free-throw gaze: increased total and mean fixation frequency (both *Z* = 2.71, *p* = 0.006) and decreased mean fixation duration (Z = 2.41, *p* = 0.016); total fixation duration non-significant. Accuracy was negatively related to fixation frequency and positively related to mean fixation duration in free throws.

Table 4 summarizes the basketball performance outcomes reported across the included studies, focusing on decision-making in video-based tasks and shooting accuracy under different fatigue and intervention conditions. It contrasts the effects of mental fatigue induced by a sport-specific videogame, with and without anodal tDCS, on perceptual-cognitive decision-making (Fortes et al., 2022, 2025), and the impact of graded physiological fatigue and attentional focus strategies on free-throw performance (Luo et al., 2025). It also synthesizes how cycling at varying intensities (Wilson et al., 2018) and a brief, basketball-specific exertion test (Zwierko et al., 2018) influence free-throw and jump-shot accuracy.

**TABLE 4:** Basketball performance outcomes (decision-making & shooting).

Study	Main findings	Effect estimates and direction of effect
Fortes et al. (2022)	Basketball decision-making task (5v5 video clips, verbal choice of best option). Outcomes: decision accuracy (%) and response time (ms). After mental-fatigue induction, accuracy decreased and response time increased under sham, whereas performance was maintained under anodal transcranial direct current stimulation (a-tDCS).	For decision-making: significant Condition × Time interactions for both accuracy and response time (e.g., response time F≈61.2, *p* = 0.001, partial η^2^≈0.25), indicating that mental fatigue impaired performance only in sham, with a-tDCS attenuating these decrements.
Fortes et al. (2025)	Basketball decision-making task (same 5v5 video task). Outcomes: decision accuracy (%) and response time (ms). Post-intervention, accuracy decreased and response time increased in videogame [VID], whereas performance remained stable in control.	Decision accuracy: Condition × Time interaction *F*(1, 15) = 8.82, *p* = 0.003, partial η^2^ = 0.08—accuracy decreased by ∼6.4% only in VID, no change in CON. Response time: Condition × Time *F*(1, 15) = 10.04, *p* = 0.001, partial η^2^ = 0.13—response time increased by ∼14.6% only in VID.
Luo et al. (2025)	Free-throw shooting accuracy: 20 free throws per focus × fatigue condition, outcome = accuracy (%). Means ± SD: No fatigue—External 82.0 ± 9.8 vs. Internal 75.0 ± 9.0; Moderate—External 78.0 ± 10.2 vs. Internal 70.0 ± 11.5; Severe—External 65.0 ± 9.1 vs. Internal 50.0 ± 10.3. Accuracy declined with increasing fatigue and was consistently higher under external vs. internal focus, especially at severe fatigue (15 percentage-point difference).	Repeated-measures ANOVA: main effect of focus *F*(1, 29) = 8.15, *p* = 0.008, partial η^2^ = 0.22 (external > internal); main effect of fatigue *F*(2, 58) = 26.32, *p* < 0.001, partial η^2^ = 0.48 (accuracy decreases with fatigue); Focus × Fatigue interaction *F*(2, 58) = 4.27, *p* = 0.018, partial η^2^ = 0.13. Simple-effects tests show a large benefit of external focus under severe fatigue [*t*(29) = 3.15, *p* = 0.003, *d* = 1.54], with smaller or non-significant effects under lower fatigue.
Wilson et al. (2018)	Free-throw performance: 10 free throws per block, scored 0–5 per shot (max 50 per block), plus % made. For SEV, mean score decreased from 32 ± 5 to 26 ± 5 /50 (∼19% drop); for HEV, 29 ± 5 to 26 ± 6 /50 (trend); REST and MOD scores remained roughly stable (exact means not fully tabulated). Success % showed no significant changes in any condition.	ANOVA revealed a significant Condition × Time interaction for free-throw score: *F*(3, 27) = 5.41, *p* = 0.005, with a significant decrease only in SEV (*p* = 0.006) and a trend in HEV (*p* = 0.093); REST and MOD unchanged. Main effect of time *F*(1, 9) = 25.88, *p* = 0.001.
Zwierko et al. (2018)	Free-throw accuracy (%): pre-S2P60 69.23% vs. post-S2P60 61.54% (small, non-significant decrease). Jump-shot accuracy during S2P60: 48.37%. Overall free-throw accuracy across test: 65.38%. Physical exertion induced only a modest reduction in free-throw accuracy, with larger changes observed in gaze behavior.	Statistical tests indicated no significant pre–post difference in free-throw accuracy (*p* > 0.05).

Table 5 focuses on the manipulation checks and fatigue-related markers used to validate the experimental interventions, distinguishing between mental and physical load. It collates the subjective ratings, eye-based indices, electroencephalography (EEG) measures and cognitive task outcomes that confirm the induction of mental fatigue in the videogame studies (Fortes et al., 2022, 2025), alongside the heart rate, rating of perceived exertion, heart-rate variability, lactate and exertion indices that characterize graded physiological fatigue and exercise-induced stress (Wilson et al., 2018; Zwierko et al., 2018; Luo et al., 2025).

**TABLE 5:** Mental/physical fatigue and manipulation checks.

Study	Manipulation check outcomes	Main findings	Effect estimates and direction
Fortes et al. (2022)	Subjective mental fatigue (VAS); eyeblink duration and pupil diameter (eye-tracking) during videogame; motivation VAS.	Eyeblink duration increased over time only in sham, indicating greater fatigue. Pupil diameter increased over time only under anodal transcranial direct current stimulation (a-tDCS), interpreted as increased cognitive control/effort with less fatigue. Mental fatigue VAS was higher post-experiment in sham vs. a-tDCS; motivation showed no condition/time effects.	Reported ANOVA results indicate significant Condition × Time interactions for eyeblink duration, pupil diameter, and mental-fatigue ratings, confirming successful induction of mental fatigue and its attenuation by a-tDCS.
Fortes et al. (2025)	Subjective mental fatigue (VAS), motivation VAS, EEG theta power (Fp1), and Stroop task performance (RT and accuracy on incongruent trials).	Mental-fatigue VAS increased only in VID, with values higher in VID than CON at post (∼286% increase from baseline). EEG theta power increased from pre to post only in VID and was higher in VID vs. CON at post. In the Stroop, response time increased only in VID and was higher in VID vs. CON at post, whereas accuracy remained unchanged.	Significant Condition × Time interactions for mental-fatigue VAS, EEG theta, and Stroop response time (all *p* ≤ 0.05, partial η^2^ in the moderate-to-large range) confirm that the videogame induced mental fatigue and impaired inhibitory control, whereas the control video did not.
Luo et al. (2025)	Heart rate (HR), rating of perceived exertion (RPE), heart-rate variability (lnRMSSD), and blood lactate measured at the end of each fatigue protocol.	Means ± SD (no → moderate → severe fatigue): HR 85 ± 8 → 150 ± 9 → 175 ± 8 bpm; RPE 7.0 ± 1.0 → 13.4 ± 1.0 → 17.2 ± 0.8; lnRMSSD 3.9 ± 0.5 → 3.2 ± 0.4 → 2.7 ± 0.4; lactate 1.6 ± 0.4 → 5.3 ± 0.8 → 9.4 ± 1.2 mmol⋅L^–1^.	Repeated-measures ANOVA showed significant effects of fatigue level on HR, RPE, HRV and lactate (all *p* < 0.001), confirming effective manipulation of physiological fatigue.
Wilson et al. (2018)	Heart rate (HR) and %HRmax during free-throw blocks pre- and post-intervention.	Post-intervention HR and %HRmax increased only in MOD, HEV, and SEV, with the greatest values in SEV (e.g. post %HRmax ≈ MOD 58 ± 4%, HEV 76 ± 6%, SEV 86 ± 5%). REST showed little change.	ANOVAs showed strong Condition, Time, and Condition × Time effects for HR and %HRmax (e.g., %HRmax: *F*(3, 27) = 147.23 for condition, *F*(1, 9) = 346.89 for time, *F*(3, 27) = 246.43 for interaction; all *p* < 0.001), indicating clearly differentiated physiological stress levels.
Zwierko et al. (2018)	Heart rate (HR) during S2P60 and repeated-sprint ability decrement (SDEC) as indices of exertion; no mental-fatigue or cognitive-effort scales.	Mean HR in the final part of S2P60 was ∼175 beats⋅min^–1^, comparable to competitive match demands; SDEC during S2P60 = 4.24 ± 1.56%.	Exertion was similar to match demands based on HR and sprint decrement.

## Discussion

The present systematic review aimed to synthesize the experimental evidence on how different forms of fatigue affect basketball players’ attentional focus, visual search behavior, and associated cognitive and physiological measures. Across only five studies, with small samples, and heterogeneous interventions the general evidence is that both mental and physical fatigue tend to impair basketball-specific decision-making and, under sufficiently high load, also shooting performance. These performance decrements are consistently accompanied by alterations in gaze behavior and indices of cognitive or physiological load. At the same time, the magnitude and consistency of effects depend strongly on the type of fatigue induced, the task demands, and moderating interventions such as brain stimulation or attentional focus.

### Attentional focus under mental and physical fatigue

Across the studies that assessed gaze behavior, there is converging evidence that both mental and physical fatigue are associated with less efficient visual search patterns. Fortes et al. (2022) reported that after the mentally fatiguing videogame, players in the sham condition showed fewer fixations and longer fixation durations when making decisions in dynamic 5-vs-5 video clips, whereas those receiving anodal transcranial direct current stimulation (a-tDCS) largely maintained their baseline visual search. The authors (Fortes et al., 2022) interpreted this pattern as a slowing and narrowing of attentional exploration under mental fatigue, consistent with an increased cognitive load that constrains the capacity to sample multiple task-relevant cues. In their behavioral trial (Fortes et al., 2025) found that the videogame-induced mental fatigue condition reduced the number of fixations during decision-making by around 9%, while fixation duration did not change significantly. This suggests that mental fatigue can reduce the breadth of visual exploration even when the temporal characteristics of individual fixations remain relatively stable. This pattern may appear to diverge from accounts in which stressors lead to more erratic, shortened fixations, however, processing efficiency theory/attentional control theory emphasize that reduced attentional control can manifest either as inefficient “scanning” or as inefficient “staring,” depending on task constraints and the balance between goal-directed control and stimulus-driven capture (Eysenck et al., 2007). In the basketball video decision tasks, mental fatigue may preferentially reduce attentional shifting and exploratory sampling, yielding fewer fixations with longer dwell times (i.e., narrowed exploration), rather than higher-frequency scanning.

The two exertion studies complement and extend these observations. Wilson et al. (2018) focused on Quiet Eye duration in free-throw shooting and showed that severe cycling to exhaustion halved QE duration, from roughly 1.5 to 0.8 s, whereas moderate and heavy cycling produced minimal or non-significant changes. Importantly, reductions in QE explained approximately one-third of the variance in changes in free-throw performance (Wilson et al., 2018). The authors (Wilson et al., 2018) discussed these findings as evidence that QE is particularly sensitive to high physiological stress, and that a shortened QE under severe fatigue reflects a breakdown in the ability to maintain goal-directed attentional control on the target throughout the critical movement phase. This aligns conceptually with Fortes et al. ’s (2022; 2025) observations since both mental and physical fatigue appear to reduce either the quantity of relevant fixations or the duration of optimal, task-focused fixations, with measurable behavioral consequences.

Zwierko et al. (2018) provided a description how exertion alters gaze in both free throws and jump shots. After the S2P60 fatigue test, free throws were characterized by more frequent fixations but shorter mean fixation durations, whereas total fixation time remained unchanged. Accuracy in free throws was negatively associated with fixation frequency and positively associated with mean fixation duration, implying that fewer but longer fixations are more beneficial, in line with the QE concept (Zwierko et al., 2018). The authors (Zwierko et al., 2018) argued that under exertion, players tended to “scan” more, perhaps reflecting instability in postural or muscular control and a less efficient visual strategy, and that this shift in gaze pattern partly offset their technical skill. These exertion-induced changes in gaze were more pronounced than the modest decline in shooting accuracy, reinforcing the notion that gaze metrics may provide more sensitive markers of fatigue than performance alone.

Although both fatigue types were associated with disrupted gaze control, the underlying mechanisms may differ. Mental fatigue plausibly impairs gaze behavior via executive-attentional deficits (reduced inhibitory control and impaired attentional shifting), producing less efficient cue sampling during decision-making (Wascher et al., 2014). By contrast, physiological fatigue may perturb gaze through exercise-induced changes in oculomotor control and sensorimotor stability, alongside central/peripheral neuromuscular constraints that alter shooting mechanics and postural control (Connell et al., 2016).

Overall, whether induced mentally or physiologically, fatigue tends to disrupt the optimal balance between fixation number and duration. Mental fatigue seems to be associated primarily with a reduced number of fixations and, in some cases, increased fixation duration, indicating a narrowed search and slower information sampling. Physical fatigue, especially when severe, reduces prolonged task-focused fixations (QE) and may increase the number of shorter fixations. Despite differences in tasks (decision-making videos vs. static free throws vs. jump shots) and measures (QE vs. general fixations), these patterns converge on the idea that fatigue degrades the efficiency of perceptual attunement to relevant cues.

### Basketball performance under mental and physical fatigue

When focusing on basketball performance outcomes, the most consistent finding is that mental fatigue impairs perceptual-cognitive decision-making, whereas physical fatigue has a more variable impact on shooting accuracy. In two crossover studies (Fortes et al., 2022, 2025) used a 60-min sport-specific videogame to induce mental fatigue and then assessed performance on a 5-vs.-5 video-based decision-making task. In the comparison between a-tDCS and sham over motion-sensitive visual cortex (Fortes et al., 2022), decision accuracy decreased and response times slowed only in the sham condition, while performance was essentially preserved when stimulation was active. In their behavioral trial (Fortes et al., 2025), the same videogame produced a clear deterioration of decision-making performance when compared with a neutral documentary condition since accuracy fell by around 6% and response times increased by about 15% only after the fatiguing videogame, with moderate to large interaction effects. Both studies (Fortes et al., 2022, 2025) therefore support the idea that sustained cognitively demanding, sport-relevant activity can meaningfully disrupt the speed–accuracy trade-off in complex decision-making, and that neuromodulation may partly buffer these effects.

Physical fatigue showed a somewhat different pattern, particularly in shooting tasks. In Wilson et al. (2018), severe exercise to exhaustion reduced free-throw scores from 32 to 26 points out of 50, whereas heavy exercise had only a borderline effect and moderate cycling or seated rest did not alter performance. Luo et al. (2025) extended this line of work by manipulating three levels of basketball-specific physiological fatigue (no, moderate and severe) and crossing them with attentional focus instructions. Free-throw accuracy decreased progressively across fatigue levels, but the decline was modulated by focus, in specific, an external focus of attention consistently yielded higher accuracy than an internal focus, with a very large benefit (65% vs. 50% accuracy) under severe fatigue. These findings align with the constrained action hypothesis discussed by Luo et al. (2025), whereby internal focus tends to disrupt automatic motor control, an effect that may be exacerbated when physiological resources are taxed.

The evidence is more nuanced when considering shorter, basketball-like exertion. Zwierko et al. (2018) reported only a small, non-significant reduction in free-throw percentage following a 60-s high-intensity jump-shot test, with jump-shot accuracy during that test remaining around 48%. The authors (Zwierko et al., 2018) emphasized that exertion had a much clearer effect on gaze behavior than on shooting success per se, suggesting that skilled players may use compensatory mechanisms to preserve accuracy despite a less efficient visual strategy. This interpretation is compatible with Wilson et al. (2018) finding that moderate and heavy exercise did not reliably impair free-throw scores, and it highlights that performance decrements may become more apparent as tasks place greater demands on decision-making (video-based tactical decisions) or attentional control (free throws under severe fatigue and internal focus), rather than on relatively well-practiced shooting alone.

Overall, the performance evidence indicates that mental fatigue likely impairs perceptual-cognitive decision-making, that physiological fatigue only likely compromises shooting when it is severe or coupled with suboptimal attentional focus, and that motor performance may be relatively robust to brief or moderate exertion.

### Mental and physiological fatigue manipulations and their validity

The two mental-fatigue studies by Fortes and colleagues (Fortes et al., 2022, 2025) used a 60-min basketball videogame as the primary manipulation, complemented by a set of manipulation checks. In Fortes et al. (2022), increases in eyeblink duration, mental-fatigue visual analog scale (VAS) scores, and changes in pupil diameter over time were all consistent with heightened fatigue during the sham condition, whereas a-tDCS appeared to modulate these markers in a manner consistent with reduced fatigue and enhanced cognitive control. In Fortes et al. (2025), mental-fatigue VAS ratings, frontal EEG theta power, and Stroop-task response times all worsened only after the videogame condition and not after the control documentary, reinforcing the interpretation that the videogame induced a distinct mental-fatigue state with cognitive consequences.

The physical-fatigue manipulations were also supported by clear manipulation checks, but they capture a somewhat different dimension of load. Luo et al. (2025) implemented a graded, basketball-specific fatigue protocol and validated it via heart rate, RPE, HRV, and blood lactate. All markers displayed stepwise increases in strain or decreases in vagal tone from no to moderate to severe fatigue, indicating distinct physiological states. Wilson et al. (2018) used cycling at different intensities, confirming condition separation through heart rate and %HRmax, with large condition and time effects. In Zwierko et al. (2018), exertion was inferred from high heart rates (∼175 bpm) and sprint decrement during the S2P60 test, again suggestive of substantial physical load comparable to match conditions. Unlike the mental-fatigue trials, none of these studies included cognitive or self-reported mental-fatigue measures (Fortes et al., 2022, 2025), so it is not possible to determine the extent to which physical exertion also induced subjective or cognitive fatigue.

Across domains, an important issue is that mental and physical fatigue have been studied largely in isolation, with different tasks and markers, making it difficult to disentangle their specific and shared mechanisms. Fortes et al. (2022, 2025) interpreted their data through a primarily cognitive lens, emphasizing impaired inhibitory control and altered attentional resource allocation as mediators of poorer decision-making. Luo et al. (2025) and Wilson et al. (2018) focused on how physiological strain disrupts motor control and attentional focus, while Zwierko et al. (2018) highlighted the sensitivity of gaze behavior to exertion even when shooting accuracy is relatively preserved. Future work could profit from combining both types of manipulation checks in the same experiments, in order to map more precisely how overlapping dimensions of “fatigue” influence perception, cognition and action in basketball.

### Limitations of the current evidence base

The small number of eligible studies and their modest sample sizes are major constraints on the strength of inference. Across the five experiments, participant numbers range from 10 to 30 players, and all samples consist of male basketball athletes. All studies recruited relatively homogeneous groups in terms of sex, sport, and competitive level, which enhances internal validity but limits generalizability to female players, youth athletes, recreational participants, and other competitive contexts. None of the included works examined positional differences, playing style, or inter-individual variability in fatigue susceptibility, and several studies relied on convenience samples from single teams or institutions.

Publication bias is a further concern. With only five eligible studies identified and a predominance of statistically significant findings, the probability of selective publication cannot be excluded, and formal small-study assessments are not informative at this sample size. This concern is consistent with broader meta-research in mental fatigue literatures indicating substantial risk of publication bias and uncertainty in adjusted effect estimates (Holgado et al., 2023).

There are also methodological concerns related to design and risk of bias. Although Fortes et al. (2022) used a double-blind crossover design with sham stimulation, other studies did not clearly describe blinding procedures or allocation concealment. Crossover and within-subject designs, as used by Fortes et al. (2025), Wilson et al. (2018), and Luo et al. (2025), are efficient in small samples but susceptible to carry-over and learning effects, particularly when the washout periods, order randomization, and practice control are not fully detailed. None of the studies reported preregistration of protocols or prospective power calculations, and selective reporting cannot be excluded, especially where multiple outcomes (performance, gaze, physiological and cognitive indices) were measured. The absence of intention-to-treat analyses or handling of missing data is another common omission, although attrition appeared low.

Ecological validity is another important limitation. Mental fatigue manipulations were based on prolonged exposure to a basketball videogame or neutral documentaries, while physical fatigue was induced by cycling, incremental drills, or brief high-intensity shooting protocols. These manipulations likely altered physiological and subjective indices, but they only approximate the complexity of real match play, where technical, tactical, emotional, and social demands interact over longer time scales. Finally, there are conceptual limitations in how “fatigue” is operationalized. The current evidence base rarely assesses these dimensions simultaneously or attempts to disentangle their relative contributions. This limits our ability to understand whether changes in gaze and performance are driven predominantly by cognitive overload, physiological strain, or their interaction.

### Main gaps in knowledge

Several substantive gaps emerge from this review. First, research on mental fatigue in basketball remains scarce and concentrated in a single program of work. Both studies by Fortes et al. (2022, 2025) use very similar manipulations and tasks and involve professional male players from comparable contexts. While they provide important initial evidence that sport-specific videogame exposure can impair decision-making and alter visual search, the generality of these findings across different playing levels, sexes, age groups, and cultural environments remains unknown.

A second gap concerns the integration of mental and physical fatigue. Luo et al. (2025), Wilson et al., 2018) and Zwierko et al. (2018) show clear effects of physiological load on shooting performance and gaze behavior, yet none of these studies include subjective mental-fatigue measures or cognitive tasks alongside physiological markers. Conversely, the mental-fatigue experiments rely on cognitive and neurophysiological checks but do not systematically quantify concurrent physical strain. This separation makes it difficult to determine whether similar gaze and performance alterations observed across studies share a common mechanism. There is a need for studies that jointly manipulate and assess both mental and physical load, allowing the effects of each to be compared, combined, and modeled within the same experimental framework.

Another major gap is the lack of longitudinal and match-based data. All included studies are acute experiments that manipulate fatigue in a single session. None track chronic exposure to training and competition load or examine whether players adapt their visual strategies and decision-making under repeated fatigue exposures over weeks or seasons. There is also no evidence on how fatigue-related decrements relate to injury risk, tactical errors, or team outcomes during real games. Integrating wearable technologies, positional tracking, eye-tracking in live or simulated matches, and subjective and objective load measures could provide a much richer understanding of how fatigue fluctuates and affects performance in ecologically meaningful settings.

Therefore, future studies should employ designs that jointly quantify cognitive and physiological load. Recommended approaches include dual-task paradigms pairing basketball-specific shooting/decision-making with validated attention tasks to index decrements in vigilance and executive control, and composite biomarker panels combining central measures with peripheral/physiological markers to dissociate fatigue modalities within the same protocol.

## Conclusion

In summary, this systematic review suggests that mental fatigue (induced via prolonged cognitively demanding, sport-relevant tasks) most consistently impairs perceptual–cognitive decision-making and alters visual search behavior, whereas physiological fatigue shows more variable effects on shooting performance, with clearer decrements and shorter quiet eye durations emerging primarily under severe exercise-induced stress and/or suboptimal attentional focus. Attentional focus strategies appear to moderate fatigue-related shooting decrements, and preliminary evidence from a small number of studies suggests that neuromodulation may attenuate mental-fatigue-related impairments in perceptual–cognitive performance. However, this evidence is currently dominated by a single research line and requires independent replication in larger preregistered trials. Larger, methodologically rigorous trials that integrate multiple fatigue modalities and outcome domains, and that include more diverse player populations and ecologically valid tasks, are needed before firm conclusions about fatigue and perceptual–cognitive performance in basketball can be drawn.

## Data Availability

The original contributions presented in this study are included in this article/supplementary material, further inquiries can be directed to the corresponding author.

## References

[B1] Angius L. Merlini M. Hopker J. Bianchi M. Fois F. Piras F. (2022). Physical and mental fatigue reduce psychomotor vigilance in professional football players. *Int. J. Sports Physiol. Perform.* 17 1391–1398. 10.1123/ijspp.2021-0387 35477898

[B2] Bourdas D. I. Travlos A. K. Souglis A. Gofas D. C. Stavropoulos D. Bakirtzoglou P. (2024). Basketball fatigue impact on kinematic parameters and 3-point shooting accuracy: Insights across players’ positions and cardiorespiratory fitness associations of high-level players. *Sports* 12:63. 10.3390/sports12030063 38535726 PMC10974731

[B3] Cao S. Geok S. K. Roslan S. Sun H. Lam S. K. Qian S. (2022). Mental fatigue and basketball performance: A systematic review. *Front. Psychol.* 12:819081. 10.3389/fpsyg.2021.819081 35082736 PMC8784842

[B4] Chinzara T. T. Buckingham G. Harris D. J. (2022). Transcranial direct current stimulation and sporting performance: A systematic review and meta-analysis of transcranial direct current stimulation effects on physical endurance, muscular strength and visuomotor skills. *Eur. J. Neurosci.* 55 468–486. 10.1111/ejn.15540 34904303

[B5] Chua L.-K. Jimenez-Diaz J. Lewthwaite R. Kim T. Wulf G. (2021). Superiority of external attentional focus for motor performance and learning: Systematic reviews and meta-analyses. *Psychol. Bull.* 147 618–645. 10.1037/bul0000335 34843301

[B6] Connell C. J. W. Thompson B. Kuhn G. Claffey M. P. Duncan S. Gant N. (2016). Fatigue related impairments in oculomotor control are prevented by caffeine. *Sci. Rep.* 6:26614. 10.1038/srep26614 27222342 PMC4879569

[B7] Dong L. Pageaux B. Romeas T. Berryman N. (2022). The effects of fatigue on perceptual-cognitive performance among open-skill sport athletes: A scoping review. *Int. Rev. Sport Exerc. Psychol.* 17 1170–1221. 10.1080/1750984X.2022.2135126

[B8] Eysenck M. W. Derakshan N. Santos R. Calvo M. G. (2007). Anxiety and cognitive performance: Attentional control theory. *Emotion* 7 336–353. 10.1037/1528-3542.7.2.336 17516812

[B9] Fortes L. S. Ferreira M. E. C. Faro H. Penna E. M. Almeida S. S. (2022). Brain stimulation over the motion-sensitive midtemporal area reduces deleterious effects of mental fatigue on perceptual-cognitive skills in basketball players. *J. Sport Exerc. Psychol.* 44 272–285. 10.1123/jsep.2021-0281 35613846

[B10] Fortes L. S. Lima-Junior D. Barbosa B. T. Faro H. K. C. Ferreira M. E. C. Almeida S. S. (2025). Effect of mental fatigue on decision-making skill and visual search behaviour in basketball players: An experimental and randomised study. *Int. J. Sport Exerc. Psychol.* 23 1–20. 10.1080/1612197X.2022.2058055

[B11] Habay J. Van Cutsem J. Verschueren J. De Bock S. Proost M. De Wachter J. (2021). Mental fatigue and sport-specific psychomotor performance: A systematic review. *Sports Med.* 51 1527–1548. 10.1007/s40279-021-01429-6 33710524

[B12] Higgins J. P. T. Savović E. Page M. J. Sterne J. A. C. (2020). Revised Cochrane risk of bias tool for randomized trials (RoB 2): Additional considerations for crossover trials. *Cochrane Methods* 1–6.

[B13] Holgado D. Mesquida C. Román-Caballero R. (2023). Assessing the evidential value of mental fatigue and exercise research. *Sports Med.* 53 2293–2307. 10.1007/s40279-023-01926-w 37682411 PMC10687172

[B14] Lebeau J.-C. Liu S. Sáenz-Moncaleano C. Sanduvete-Chaves S. Chacón-Moscoso S. Becker B. J. (2016). Quiet eye and performance in sport: A meta-analysis. *J. Sport Exerc. Psychol.* 38 441–457. 10.1123/jsep.2015-0123 27633956

[B15] Li S. Luo Y. Cao Y. Li F. Jin H. Mi J. (2025). Changes in shooting accuracy among basketball players under fatigue: A systematic review and meta-analysis. *Front. Physiol.* 16:1435810. 10.3389/fphys.2025.1435810 40078369 PMC11897034

[B16] Lohse K. R. Sherwood D. E. (2011). Defining the focus of attention: Effects of attention on perceived exertion and fatigue. *Front. Psychol.* 2:332. 10.3389/fpsyg.2011.00332 22102843 PMC3214735

[B17] Luo Y. Cao Y. Li S. Shi Y. Chen P. (2025). Effects of physiological fatigue on basketball shooting performance: The moderating role of attentional focus. *Front. Psychol.* 16:1593182. 10.3389/fpsyg.2025.1593182 41089649 PMC12517065

[B18] Lyons M. Al-Nakeeb Y. Nevill A. (2006). The impact of moderate and high intensity total body fatigue on passing accuracy in expert and novice basketball players. *J. Sports Sci. Med.* 5 215–227. 24259994 PMC3827563

[B19] Marcora S. M. Staiano W. Manning V. (2009). Mental fatigue impairs physical performance in humans. *J. Appl. Physiol.* 106 857–864. 10.1152/japplphysiol.91324.2008 19131473

[B20] Martinho D. V. Clemente F. M. Gomez M. -Á. Rebelo A. Field A. Santos C. C. (2025). Physical, physiological, technical and tactical responses according to playing position in male basketball: A systematic scoping review. *J. Hum. Kinet.* 96 5–35. 10.5114/jhk/203326 40453900 PMC12121883

[B21] McInnes S. E. Carlson J. S. Jones C. J. McKenna M. J. (1995). The physiological load imposed on basketball players during competition. *J. Sports Sci.* 13 387–397. 10.1080/02640419508732254 8558625

[B22] Noakes T. D. (2007). The central governor model of exercise regulation applied to the marathon. *Sports Med.* 37 374–377. 10.2165/00007256-200737040-00026 17465612

[B23] Noakes T. D. (2012). Fatigue is a brain-derived emotion that regulates the exercise behavior to ensure the protection of whole body homeostasis. *Front. Physiol.* 3:82. 10.3389/fphys.2012.00082 22514538 PMC3323922

[B24] Nuño A. Chirosa I. J. van den Tillaar R. Guisado R. Martín I. Martinez I. (2016). Effects of fatigue on throwing performance in experienced team handball players. *J. Hum. Kinet.* 54 103–113. 10.1515/hukin-2016-0039 28031762 PMC5187964

[B25] Page M. J. McKenzie J. E. Bossuyt P. M. Boutron I. Hoffmann T. C. Mulrow C. D. (2021). The PRISMA 2020 statement: An updated guideline for reporting systematic reviews. *BMJ* 372:n71. 10.1136/bmj.n71 33782057 PMC8005924

[B26] Petway A. J. Freitas T. T. Calleja-González J. Medina Leal D. Alcaraz P. E. (2020). Training load and match-play demands in basketball based on competition level: A systematic review. *PLoS One* 15:e0229212. 10.1371/journal.pone.0229212 32134965 PMC7058381

[B27] Piras A. (2024). The timing of vision in basketball three-point shots. *Front. Psychol.* 15:1458363. 10.3389/fpsyg.2024.1458363 39539305 PMC11558341

[B28] Sirnik M. Erc̆ulj F. Rošker J. (2022). Research of visual attention in basketball shooting: A systematic review with meta-analysis. *Int. J. Sports Sci. Coach.* 17 1195–1210. 10.1177/17479541221075740

[B29] Slawinski J. Louis J. Poli J. Tiollier E. Khazoom C. Dinu D. (2018). The effects of repeated sprints on the kinematics of 3-point shooting in basketball. *J. Hum. Kinet.* 62 5–14. 10.1515/hukin-2017-0156 29922372 PMC6006537

[B30] Smith M. R. Zeuwts L. Lenoir M. Hens N. De Jong L. M. S. Coutts A. J. (2016). Mental fatigue impairs soccer-specific decision-making skill. *J. Sports Sci.* 34 1297–1304. 10.1080/02640414.2016.1156241 26949830

[B31] Sterne J. A. Hernán M. A. Reeves B. C. Savović J. Berkman N. D. Viswanathan M. (2016). ROBINS-I: A tool for assessing risk of bias in non-randomised studies of interventions. *BMJ* 355:i4919. 10.1136/bmj.i4919 27733354 PMC5062054

[B32] Stojanovic E. Stojiljkovic N. Scanlan A. T. Dalbo V. J. Berkelmans D. M. Milanovic Z. (2018). The activity demands and physiological responses encountered during basketball match-play: A systematic review. *Sports Med.* 48 111–135. 10.1007/s40279-017-0794-z 29039018

[B33] Sun H. Soh K. G. Roslan S. Wazir M. R. W. N. Soh K. L. (2021). Does mental fatigue affect skilled performance in athletes? A systematic review. *PLoS One* 16:e0258307. 10.1371/journal.pone.0258307 34648555 PMC8516214

[B34] Trecroci A. Boccolini G. Duca M. Formenti D. Alberti G. (2020). Mental fatigue impairs physical activity, technical and decision-making performance during small-sided games. *PLoS One* 15:e0238461. 10.1371/journal.pone.0238461 32903263 PMC7480836

[B35] Van Cutsem J. Marcora S. De Pauw K. Bailey S. Meeusen R. Roelands B. (2017). The effects of mental fatigue on physical performance: A systematic review. *Sports Med.* 47 1569–1588. 10.1007/s40279-016-0672-0 28044281

[B36] Vickers J. N. Causer J. Vanhooren D. (2019). The role of quiet eye timing and location in the basketball three-point shot: A new research paradigm. *Front. Psychol.* 10:2424. 10.3389/fpsyg.2019.02424 31736825 PMC6836760

[B37] Wascher E. Rasch B. Sänger J. Hoffmann S. Schneider D. Rinkenauer G. (2014). Frontal theta activity reflects distinct aspects of mental fatigue. *Biol. Psychol.* 96 57–65. 10.1016/j.biopsycho.2013.11.010 24309160

[B38] Wilson M. R. Webb A. Wylie L. J. Vine S. J. (2018). The quiet eye is sensitive to exercise-induced physiological stress. *Prog. Brain Res.* 240 35–52. 10.1016/bs.pbr.2018.08.008 30390839

[B39] Wu C.-H. Zhao Y.-D. Yin F.-Q. Yi Y. Geng L. Xu X. (2024). Mental fatigue and sports performance of athletes: Theoretical explanation, influencing factors, and intervention methods. *Behav. Sci.* 14:1125. 10.3390/bs14121125 39767266 PMC11673376

[B40] Zwierko T. Popowczak M. Woźniak J. Rokita A. (2018). Visual control in basketball shooting under exertion conditions. *J. Sports Med. Phys. Fitness* 58 1544–1553. 10.23736/S0022-4707.17.07522-3 28745471

